# An Analysis of Microwave Ablation Parameters for Treatment of Liver Tumors from the 3D-IRCADb-01 Database

**DOI:** 10.3390/biomedicines10071569

**Published:** 2022-07-01

**Authors:** Marija Radmilović-Radjenović, Nikola Bošković, Martin Sabo, Branislav Radjenović

**Affiliations:** 1 Institute of Physics, University of Belgrade, Pregrevica 118, 11080 Belgrade, Serbia; nikolab@ipb.ac.rs (N.B.); bradjeno@ipb.ac.rs (B.R.); 2 Faculty of Informatics and Information Technologies, Slovak University of Technology in Bratislava, Ilkovicova 2, 84216 Bratislava, Slovakia; martin.sabo@stuba.sk

**Keywords:** liver tumor, microwave ablation, ablation zone, necrotic tissue

## Abstract

Simulation techniques are powerful tools for determining the optimal conditions necessary for microwave ablation to be efficient and safe for treating liver tumors. Owing to the complexity and computational resource consumption, most of the existing numerical models are two-dimensional axisymmetric models that emulate actual three-dimensional cancers and the surrounding tissue, which is often far from reality. Different tumor shapes and sizes require different input powers and ablation times to ensure the preservation of healthy tissues that can be determined only by the full three-dimensional simulations. This study aimed to tailor microwave ablation therapeutic conditions for complete tumor ablation with an adequate safety margin, while avoiding injury to the surrounding healthy tissue. Three-dimensional simulations were performed for a multi-slot microwave antenna immersed in two tumors obtained from the 3D-IRCADb-01 liver tumors database. The temperature dependence of the dielectric and thermal properties of healthy and tumoral liver tissues, blood perfusion, and water content are crucial for calculating the correct ablation time and, thereby, the correct ablation process. The developed three-dimensional simulation model may help practitioners in planning patient-individual procedures by determining the optimal input power and duration of the ablation process for the actual shape of the tumor. With proper input power, necrotic tissue is placed mainly in the tumor, and only a small amount of surrounding tissue is damaged.

## 1. Introduction

Liver cancer, also known as hepatic cancer, arises because of the abnormal growth of cells inside the liver [1,2,3,4,5]. It may originate in the liver from hepatocytes, bile duct epithelium, or mesenchymal tissue (primary) or spread to the liver from primary cancer developed elsewhere in the body (secondary) [6,7,8,9,10]. The most prevalent type of liver cancer is Hepatocellular carcinoma (HCC) or hepatoma with a median survival time of fewer than six months if untreated, and a five-year survival rate of only 5–9% from the time of diagnosis [11,12]. Since HCC is the sixth most common cancer and the second most common cause of cancer mortality worldwide, establishing an efficient treatment for this type of cancer has never been more urgent [13,14,15,16,17,18].

Treatments for liver cancer are strongly dictated by the tumor stage and the extent of the underlying liver disease, in addition to the patient’s overall age and health [19,20,21,22]. Despite recent advances in therapeutic options for liver cancer, it remains one of the most difficult cancers to treat [23]. Among the various applicable procedures, microwave ablation has proven to be an effective minimally invasive procedure for curing liver malignancies [24,25,26,27,28]. HCC ablation is defined by the Barcelona Clinic Liver Cancer (BCLC) algorithm that should be applied to each patient individually [29]. The success rate for eliminating small liver tumors in patients treated with MWA is greater than 85% [30]. MWA is also used for the treatment of liver metastases from colorectal cancer [31].

MWA is a widely used thermal ablation modality for eradicating malignant cells with minimal damage to the surrounding tissues [32,33,34]. It consists in focusing an energy source in the target zone (the tumoral tissue) causing tumor destruction. Some benefits of MWA include a large zone of treated tissue, short treatment duration, and less susceptibility to the heat sink effect generated by the cooling effect of blood flow. One of the main problems with ablation therapy is tumor recurrence and the exact prediction of tissue temperature requiring ablation zone monitoring. CT thermography allows the measurement of temperature non-invasively during ablation and is crucial to achieving a successful ablation with completely devitalized tumors [35]. MWA destroys tumors using one or more antennae as the source of the microwave fields, which leads to the frictional heating of water molecules in the soft tissues around the field source. A key element in MWA treatments is the microwave antenna (MW), which delivers energy and provides lethal temperature rise, resulting in cell death in the ablation zone [36]. Advanced antenna designs are based on three different mechanisms: thermal, field, and wavelength control [37]. Recently, a compact, multi-slot coaxial antenna was developed to achieve the required ablation zone and suitable impedance matching to the target tumoral tissue without damaging the surrounding healthy tissues [38].

Nowadays, thermal therapy has evolved into a very important topic in medicine, and many studies on the application of heat transfer to living tissues have been carried out in the last few decades, including cancer tumor treatment, drug delivery [39], or pain relief [40]. Numerical studies may have a great impact on patient care by creating predictive models from procedural planning to execution [41,42,43]. Moreover, the lack of experimentation in this field underlines mathematical models even more significantly. In this context, understanding the physics behind thermal therapy has a key role in modeling heat transfer in thermal therapies, to develop more and more accurate tissue and process models. Every mathematical model for the simulation of MWA must contain three fundamental components: the antenna probe, heat distribution in the tissue, and the effect of heat on tumor cells and their destruction. All these components depend on a diversity of material parameters, which, in turn, depend on the various states of the tissue characteristics of the individual patient. 

Recently, MWA has been analyzed using a more complex heat transfer model based on the porosity concept that leads to two bioheat equations for tissue and blood temperatures [44]. The shape of the tumor is supposed to be spherical. Although it seems to be more realistic, the problem is that such an approach introduces a new set of parameters, whose values are very difficult to determine precisely. In an earlier reference [45], an effort to include the effects of tissue deformation during MWA was made, but only for an in vitro experiment with the liver (without tumor) under specific mechanical conditions, far from realistic MWA procedures. Both references present 2D axisymmetric calculations only. Most of the existing numerical models of MWA are mainly two-dimensional (2D) axis-symmetric, assuming a homogeneous medium and reducing the problem from three-dimensional (3D) to 2D [43,46,47]. 

Considering that each liver tumor is different, treatment options must be chosen on an individualized basis, depending on the tumor size and shape. The primary goal of this study was to demonstrate that 3D simulation is an ideal technique for MWA planning adjusted for each patient. For this purpose, we used a full 3D model of the MWA developed and tested using the COMSOL Multiphysics simulation platform [48,49,50]. Simulations were performed for a compact 10-slot coaxial antenna operating at 2.45 GHz inserted into realistic models of two tumors labeled as 1.07 and 1.03 in the 3D-IRCADb-01 liver tumors database [51]. The optimal input power and duration of the ablation process were individually determined for each tumor. based on the time evolution of the iso-contours, temperature distribution, and degree of tissue destruction.

## 2. Materials and Methods

**Figure 1:**
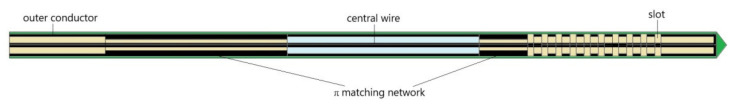
Schematic view of the 10-slot microwave antenna with an impedance π-matching network. The conducting material, Teflon, air, and dielectric are represented by black, green, light blue, and light brown, respectively. The width of the slot is 0.6 mm with a spacing of 0.8 mm between adjacent slots.

For this study, calculations were performed for a compact 10-slot microwave antenna, schematically shown in Figure 1 and described in detail in Ref. [48]. A compact 10-slot microwave antenna with an impedance π-matching network was designed to create predictable, optimal heating patterns with shorter ablation times and lower input powers, compared to previously developed antennae [52,53,54]. The required ablation shape was achieved by adopting the distance between the adjacent slots and the number of the slots. The finely tuned impedance π-matching network provides optimal ablation zones with minimal damage to surrounding healthy tissues. The disadvantage of such an antenna could be a relatively complicated construction, although it is compensated by its excellent features—optimal heating pattern and low overheating of healthy tissue.

**Figure 2:**
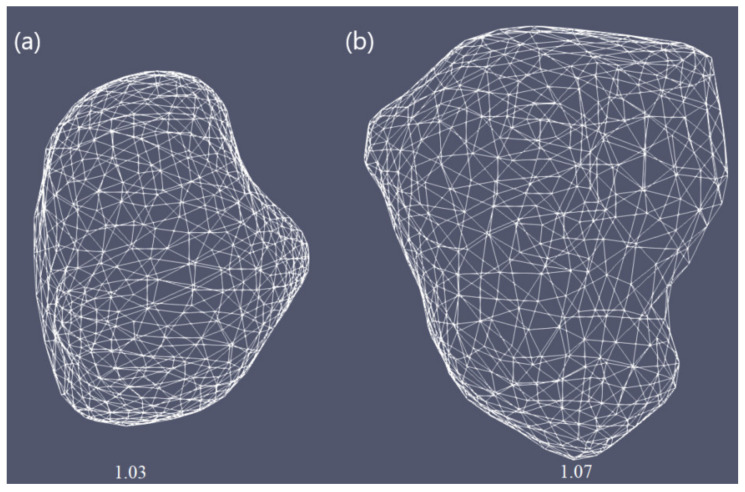
Three-dimensional simulation models corresponding to two liver tumors (triangulated surfaces) labeled as (**a**) 1.07 and (**b**) 1.03 in the 3D-IRCADb-01 database that contains several sets of CT scans of the patients [51].

To demonstrate the importance of finding the optimal conditions for each patient individually, simulations were performed for two real tumors taken from the 3D-IRCADb-01 database [51]. This database includes several sets of CT scans of patients manually segmented by clinical experts [55]. In this study, simulations were carried out for tumors denoted by 1.07 (1.74 cm × 1.53 cm × 2.10 cm) and 1.03 (1.78 cm × 1.97 cm × 2.27 cm) in the 3D-IRCADb-01 liver tumors database [51]. The sizes and shapes of both cancers are shown in Figure 2. 

The simulation model was composed of the coupled electromagnetic field and heat-transfer equations solved by the 3D finite elements method (FEM), with all details of multi-slot antenna design and properties of healthy and tumoral tissues. Our 3D model was generated using the COMSOL Multiphysics FEM-based simulation platform [45]. Sinc the developed 3D model was completely described in a previous paper [48], we shall not attempt to repeat it here, except for the governing equations. The propagation of microwaves in the tissue by an antenna is expressed as [48,56]:

(1) $$\nabla^{2}E-\mu_{r}k_{0}^{2}\left(\varepsilon_{r}-\frac{j\sigma }{\omega \varepsilon_{0}}\right)E=0,$$

where ***E*** is the vector of the electric field and *ω* is the angular frequency. The value *k*_0_ = *ω*/*c*_0_ is the vacuum propagation constant, and *ε*_0_ is the vacuum dielectric constant. The electrical conductivity, relative permittivity, and permeability of the tissue are denoted by *σ,* ε*_r_,* and *μ_r_*, respectively. The electric field was calculated in the dielectric, healthy tissue, and tumor regions, with appropriate boundary conditions. On conducting surfaces, PEC conditions were imposed (the tangential component of the field was set to zero). On the computational zone boundaries (outer cylinder surfaces) the first order absorbing boundary conditions were used. The input power is connected to the antenna through the coaxial port on its top.

The Pennes bioheat equation describes the heat transfer during MWA [57]:

(2) $$\rho c_{\mathrm{eff}}\frac{\partial T}{\partial t}=\nabla \cdot \left(k\nabla T\right)+\rho_{b}W_{b}c_{b}\left(T_{b}-T\right)+Q_{\mathrm{ext}}+Q_{m},$$

where *t* is the time, *ρ* and *T* are the density and temperature of the tissue, respectively. Values *ρ*_b_, *c*_b_, *T*_b_, and *W*_b_ are the density, heat capacity, temperature, and perfusion rate of the blood, respectively. Although the heat source from metabolism *Q*_m_ was neglected in our calculations, the external heat source *Q*_ext_ was related to coupling with the electromagnetic field. The effect of internal water evaporation in the bioheat Equation (2) was included by replacing specific heat *c* with an effective value, as described in ref. [48]:

(3) $$c_{\mathrm{eff}}=c-\frac{\alpha }{\rho }\frac{\partial W}{\partial T}$$

where *α* is the water latent heat constant equal to 2260 (kJ/kg), whereas *W*(*T*) is the water content. The temperature was calculated only in healthy and tumor regions. Initial temperature was set to 37 °C. On the outer antenna surface and computational zone, boundaries zero flux (thermal insulation) boundary conditions were used. 

The water content of liver tissue is approximately 78% water by mass; therefore, the thermal properties of the tissue are similar to those of water [58,59]. For temperatures above 100 °C, the water content of the tissue may decrease by mass owing to evaporation, causing substantial changes in tissue dielectric parameters and considerable penetration of microwaves [58]. The decrease in the dielectric properties of the tissue with increasing temperature due to evaporation was incorporated into our model according to the description in [48,60]. 

*Tissue damage* processes are regularly modeled via Arrhenius formalism defining an arbitrary function of tissue injury *Ω* as [61,62]:

(4) $$\Omega \left(t\right)=\int_{0}^{t}A\exp\left(-\frac{\Delta E}{RT}\right)dt,$$

where *A* is the frequency factor, Δ*E* is the activation energy for the irreversible damage reaction, *T* is the temperature determined at each point in the model region, and *R* is the gas constant.

The proper choice of the input power and treatment time required to achieve the optimal ablation zones strongly depends on the size and the shape of the tumor. In this study, we performed realistic modeling of the effect of MWA operating at 2.45 GHz on two liver tumors of different sizes and shapes, denoted by 1.03 and 1.07 in the 3D-IRCADb-01 database [46]. The optimal input power and duration of the ablation process were individually estimated for each tumor. The simulation conditions included the density, dielectric properties, thermal conductivity, and heat capacity of the liver tissues (healthy and tumoral), as well as the density, thermal conductivity, and heat capacity of the blood listed in Table 1. The temperature dependence of the dielectric and thermal properties of both healthy and malignant liver tissues, blood perfusion, and water content were implemented in the simulation model [48,56].

## 3. Results and Discussions

**Figure 3:**
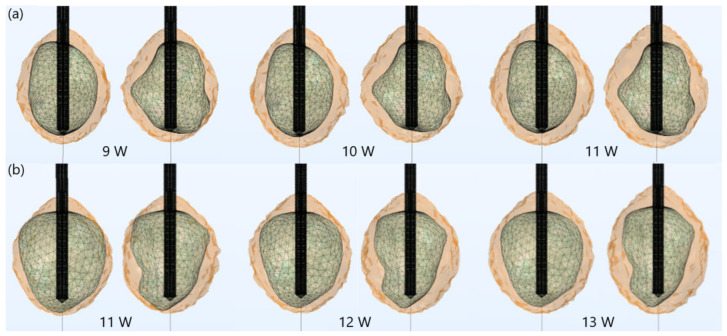
Iso-contours representing the ablated regions (solid light brown surface) after 600 s of MWA at 2.45 GHz around tumors (triangulated surface) (**a**) 1.07 [51] (for input powers of 9 W, 10 W, and 11 W) and (**b**) 1.03 [51] (for input powers of 11 W, 12 W, and 13 W).

For both tumors, the optimal value of the input power was chosen such that a very small area of healthy tissue around the tumor was damaged, based on the iso-contours shown in Figure 3. Since tumors do not have regular shapes on both sides, the front (left) and back (right) of tumors are displayed. For tumor 1.07 [51] (see Figure 3a), if 9 W was applied, tumoral tissue (backside) was not entirely removed. When the input power was 11 W, the ablation zone enclosed the entire tumor as well as a large amount of healthy tissue. The iso-contour that best fit the necrotic tissue was obtained for an input power of 10 W, leading to a treated tumor with minimal damage to the healthy surrounding tissue. However, the application of 11 W in the case of tumor 1.03 [51] (see Figure 3b) did not ensure complete malignancy (both sides). Although the entire tumor was eliminated using an input power of 13 W, the healthy tissue was significantly damaged. Therefore, the best choice of input power, for tumor 1.03 [51] was 12 W, because the whole tumor was destroyed while healthy tissue was preserved.

**Figure 4:**
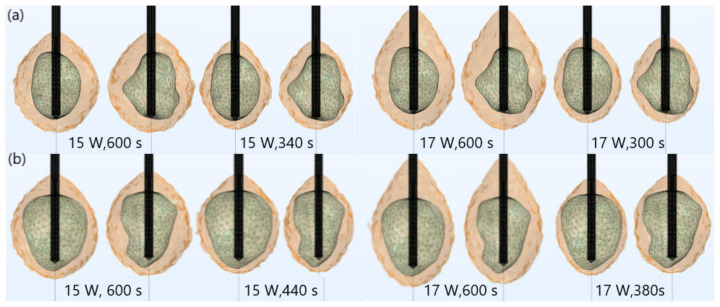
Iso-contours consisting of totally ablated regions (solid light brown surface) and tumors (triangulated surface) (**a**) 1.07 [51] and (**b**) 1.03 [51] exposed to a frequency of 2.45 GHz, an input power of 15 W and 17 W for various ablation times.

**Figure 5:**
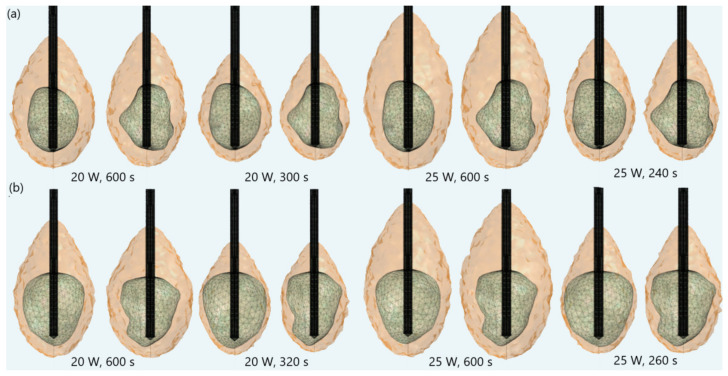
Iso-contours that include the ablated regions (solid light brown surface) around the liver tumor (triangulated surface) (**a**) 1.07 [51] and (**b**) 1.03 [51] when an input power of 20 W or 25 W is applied during various ablation times.

The importance of proper determination of the input power and ablation time is illustrated in Figure 4. If a power of 15 W or 17 W was applied for MWA of the tumor 1.07 [51] (Figure 4a), the ablation time should be shortened from 600 s to 340 s or 300 s, respectively. If tumor 1.03 [46] (Figure 4b) was treated with an input power of 15 W or 17 W, the ablation time required for complete removal of tumoral tissue should be 440 s or 380 s, respectively. Damages to healthy surrounding tissue by applying 15 W or 17 W during a shorter ablation time were similar to those obtained for 10 W (for tumor 1.07 [51]) and 12 W (for tumor 1.07 [51]) after 600 s. However, this did not necessarily mean that higher input power and shorter ablation time corresponded to the most efficient and safe MWA procedure. As can be observed from Figure 5, higher input power increased damage to surrounding healthy tissue, due to undesirable shapes of ablation zones, even if the ablation time was shorter. For powers of 20 W (for tumor 1.07 [51]) and 25 W (for tumor 1.03 [46]) the ablation zones formed were neither spherical nor predictable. They appeared to be elongated, with a greater length along the shaft of the antenna than the transverse diameter. Elongated shapes were undesirable ablation patterns that caused unavoidable damage to normal tissues, even if the ablation time was shorter. 

**Figure 6:**
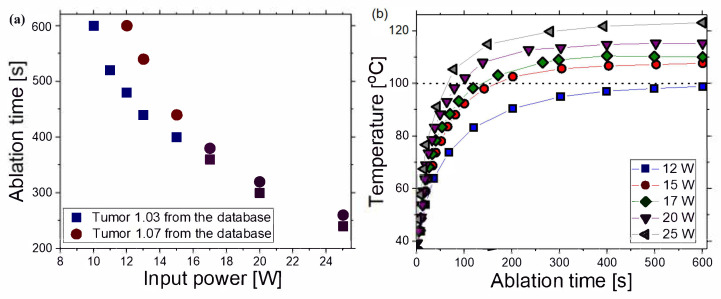
(**a**) Dependence of the ablation time on the input power for tumor 1.03 [51] (blue squares) and tumor 1.07 [51] (red circles). (**b**) Temperature as a function of the ablation time for various input power values calculated in the center of the heating zone.

The ablation times as a function of the input power for tumors 1.03 [51] and 1.07 [51] are plotted in Figure 6a. Although the ablation time decreased with increasing power for both tumors, the ablation time of tumor 1.03 [51] was systematically higher, owing to its larger size. The difference between ablation times of tumors 1.03 [51] and 1.07 [51] was approximately 25% for the power of 12 W and approximately 8% for 25 W. Controlling the power/time values allowed for changes in ablation sizes. The optimal choice of the input power and the ablation time should be made by the surgeon since it depends on medical factors. The time dependence of the temperature on the input power calculated at the point in the center of the heating zone is shown in Figure 6b. The antenna immersed in the tissue radiated energy that was absorbed and converted into thermal energy, causing an increase in the tissue temperature. Despite the input power, the temperature first rapidly increased with increasing time, and then steeply rose and reached saturation when the diffusion and heat conduction, due to blood perfusion, became significant. The obtained simulation results had similar trends as the results of measurements for liver tissue provided in Ref. [63]. 

**Figure 7:**
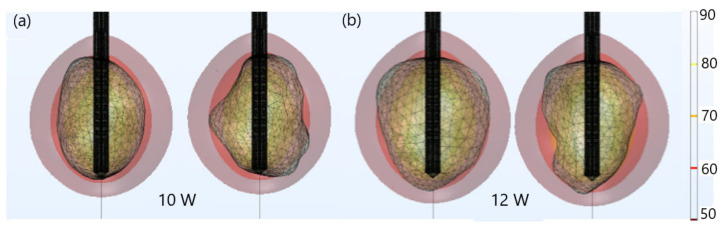
Iso-contours associated with various temperatures (solid colored surface) around tumors (triangulated surface) (**a**) 1.07 [51] (for an input power of 10 W) and (**b**) 1.03 [51] (for an input power of 12 W).

Figure 7 displays iso-contours of the temperature distribution in the tissue at *t* = 600 s for the optimal input power of 10 W and 12 W for tumors (a) 1.07 [51] and (b) 1.03 [51], respectively, chosen to affect a very small area of surrounding healthy tissue. For both tumors, iso-contours had similar near-spherical shapes. The temperature was the highest in the vicinity of the antenna slot, while it noticeably dropped as the distance from the antenna increased. The maximum values of the temperature were achieved inside the tumor regions where cancer cells were destroyed. It was reported that temperatures above 60 °C instantly destroyed all cancer cells [62], so the 60 °C isothermal contour was related to the lesion size and shape of the ablated tissue. According to previous studies [48,56], a multi-slot antenna structure enabled more localized and optimal heating distributions.

**Figure 8:**
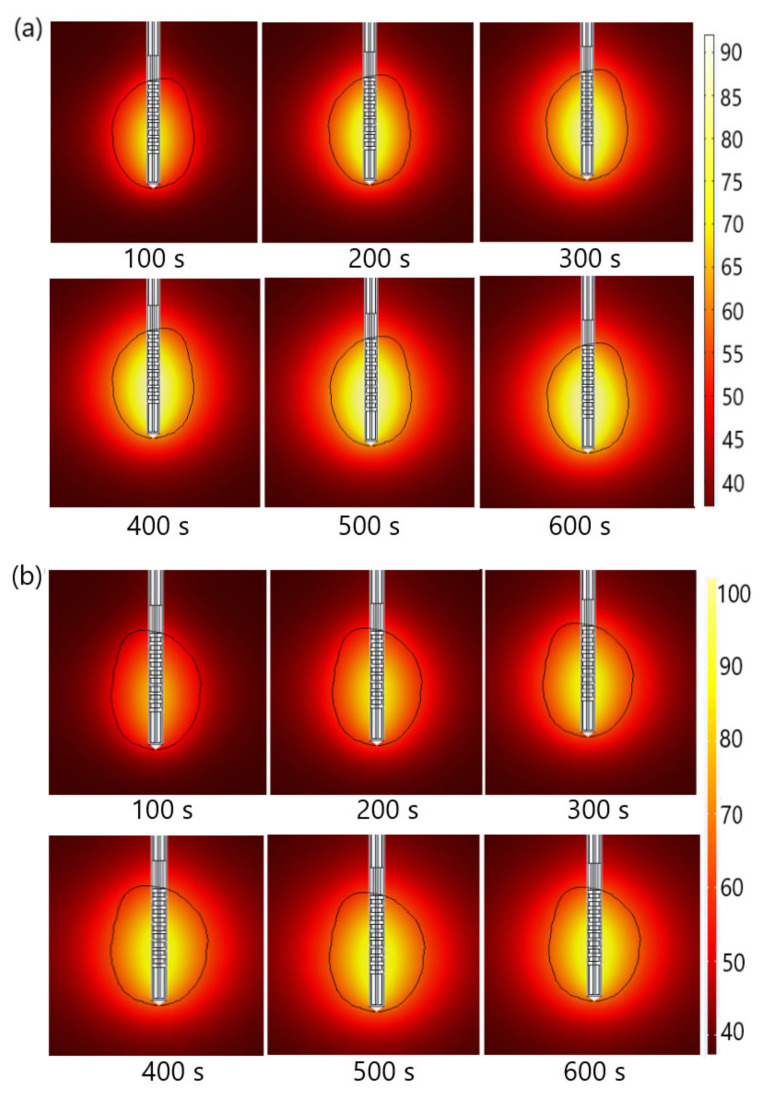
Time evolution of the temperature distribution (in °C) for tumors (**a**) 1.07 [51]) and (**b**) 1.03 [51] treated by MWA at a frequency of 2.45 GHz. The boundary of the tumor tissue is marked by a black line.

The absorbed energy was converted into thermal energy, resulting in an increase in tissue temperature. Temperature changes in tissues during MWA for tumors (a) 1.07 [51] and (b) 1.03 [51] are shown in Figure 8. The black line represents the boundary of the tumoral tissue. The temperature value was the highest close to the antenna and decreased with distance from the antenna, where the heat source became weaker. Blood perfusion restricted the extent of the heated area. The temperature increased with the ablation time and after 600 s reached a value of approximately 92 °C for tumor 1.07 [51] and 98 °C for tumor 1.03 [51]. When the temperature approached 100 °C, a boiling effect could occur in the tissues [64]. 

**Figure 9:**
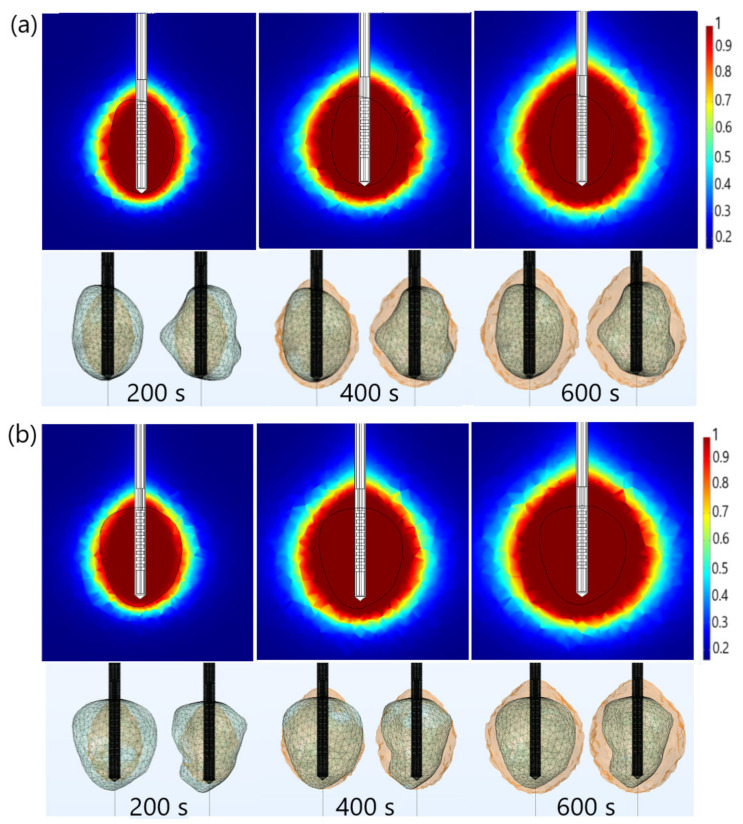
The time evolution of the necrotic tissue of tumors (**a**) 1.07 [51] and (**b**) 1.03 [51], after 200 s, 400 s, and 600 s MWA at a frequency of 2.45GHz. The upper figures show the necrotic tissue in the cut plane (x = 0). The lower figures show the front and back sides of the tumors (triangulated surfaces).

The thermal damage fraction and the time required for complete necrosis of tumors (a) 1.07 [51] and (b) 1.03 [51] are presented in Figure 9. The upper figures are obtained in the cut plane (x = 0), where the black line shows the boundary of the tumoral tissue. Regardless of the time, ablation zones were concentrated around the tip and slots of the antenna with small backward heating. The active ablation zone closest to the antenna encompassed the volume of tissue that was subjected to sufficiently high energy absorption to ensure thermal tissue. In contrast, the passive ablation zone surrounded the active zone involving the volume of tissue, which experienced a lower intensity of energy absorption [43,48]. However, the ablation zones shown in the upper figures (in the cut plane) may not accurately reflect the ablated tissue. Based on the upper figures, for example, one might conclude that the entire tumoral tissue was removed after 400 s, which was far from reality. As can be seen from the lower figures, the front and back sides of both tumors were not completely ablated after 400 s. The lower figures indicated the entire tumors would not be removed before 600 s, implying the importance of performing full 3D simulations for each tumor individually, due to tumor geometric complexity. Figure 9 indicates that correct ablation time can be determined only if calculations are performed for actual shapes of tumors. 

## 4. Conclusions

This study aimed to determine the most influential parameters (input power and ablation time) for successful tumor ablation with an adequate safety margin, while avoiding injury to the surrounding healthy tissue. To achieve this goal, full three-dimensional simulations developed within the COMSOL simulation package were used. Calculations were performed for a 10-slots antenna operating at 2.45 GHz immersed in liver tumors labeled as 1.07 and 1.03 in the database 3D-ICRADb-01 [51]. The density, thermal conductivity, and heat capacity of the liver tissues (healthy and tumoral) and blood were collected from the literature [48,56]. The temperature dependence of the dielectric and thermal properties of both healthy and malignant liver tissues, blood perfusion, and water content were included because of the importance of establishing a correct ablation process. 

The size of the ablative zone was determined by the amount of energy delivered from the microwave generator to the antenna. Higher input power than the optimal value led to significant damage to surrounding healthy tissue, due to undesirable shapes of ablation zones. The optimal values of the input power for tumors 1.07 [51] and 1.03 [51] were 10 W and 12 W, respectively, enabling optimal ablation zones concentrated around the tip and slots of the antenna, resulting in successful ablation of the tumors with minimal damage to healthy tissues. As expected, a higher power would increase temperatures and reduce the overall time to necrose tumoral tissue. For tumor 1.07 [51], the ablation times for 25 W and 10 W differed by approximately 60%. For tumor 1.03 [51], the application of 25 W would reduce the ablation time by approximately 57%, compared to that for 12 W. However, higher values of input power and shorter ablation time sometimes led to the formation of elongated ablation zones, causing significant damage to healthy tissue around the tumor. In addition, it was reported that delivery of high-power, short-duration ablation is not commonly used because of the increased risk of steam pop and thrombus formation [65]. The temperature increased with the ablation time, reaching the maximum value near the microwave antenna slots. The maximal temperature for 25 W differed by approximately 21%, 15%, 9%, and 8.5% from those calculated for 12 W, 15 W, 17 W, and 20 W, respectively. It was also demonstrated that axisymmetric calculations are not sufficient to estimate the optimal input power and ablation time. Thus, full 3D simulations, that take into account details of the tumor geometry, are needed [48,66]. 

The obtained results can be used to determine the optimal conditions for microwave ablation to be as effective as possible for treating liver tumors with minimal invasiveness and collateral damage. The developed three-dimensional predictive model of the microwave ablation procedure with all details of the tissue antenna design is a prerequisite, not only for further ablation studies but also for planning the MWA procedure for each patient individually. Regarding the limitations of the simulation model, it has to be pointed out that macroscale models of biological tissues are either based on the mixture theory of continuum mechanics or on the porous-media theory [67]. Simply put, Pennes’ bioheat equation belongs to the first group, reducing the complete tissue model to a single heat transport equation with an additional term describing heat removal by blood perfusion. Models based on porous-media theory [67,68,69] are more complex, usually containing at least two equations: one for tissue and one for blood temperature. Of course, more subtle aspects can be included (thermo-mechanical, microscopic, etc.), but in this paper, we concentrated on problems connected with full 3D modeling of antennae used in MWA procedures and realistic tumor shapes of actual patients. Finally, it has to be pointed out that before any applications, experimental validation is mandatory to verify the reliability of predictive models. 

## Figures and Tables

**Table 1:** The parameters that characterize the liver tissue (healthy and tumoral) and the blood collected from the literature [48,56] and used in the numerical simulations.

Parameter	Value
***Healthy tissue***	
Density	1079 kg/m^3^
Relative permittivity	44.3
Electric conductivity	1.8 S/m
Thermal conductivity	0.52 W/m °C
Specific heat	3540 J/kg °C
***Tumoral tissue***	
Density	1040 kg/m^3^
Relative permittivity	54.8
Electric conductivity	2 S/m
Thermal conductivity	0.57 W/m °C
Specific heat	3960 J/kg °C
***Blood***	
Density	1060 kg/m^3^
Thermal conductivity	0.5 W/m °C
Specific heat	3600 J/kg °C
Temperature	37 °C

## Data Availability

Data is contained within the article.
